# Compliant, Tough, Anti-Fatigue, Self-Recovery, and Biocompatible PHEMA-Based Hydrogels for Breast Tissue Replacement Enabled by Hydrogen Bonding Enhancement and Suppressed Phase Separation

**DOI:** 10.3390/gels8090532

**Published:** 2022-08-25

**Authors:** Hongyan Ouyang, Xiangyan Xie, Yuanjie Xie, Di Wu, Xingqi Luo, Jinrong Wu, Yi Wang, Lijuan Zhao

**Affiliations:** 1 College of Chemistry and Materials Science, Sichuan Normal University, Chengdu 610068, China; 2 State Key Laboratory of Polymer Materials Engineering, College of Polymer Science and Engineering, Sichuan University, Chengdu 610065, China

**Keywords:** breast reconstruction, PHEMA-based hydrogel, hydrogen bond enhancement, phase separation inhibition, biocompatibility

## Abstract

Although hydrogel is a promising prosthesis implantation material for breast reconstruction, there is no suitable hydrogel with proper mechanical properties and good biocompatibility. Here, we report a series of compliant and tough poly (hydroxyethyl methacrylate) (PHEMA)-based hydrogels based on hydrogen bond-reinforcing interactions and phase separation inhibition by introducing maleic acid (MA) units. As a result, the tensile strength, fracture strain, tensile modulus, and toughness are up to 420 kPa, 293.4%, 770 kPa, and 0.86 MJ/m^3^, respectively. Moreover, the hydrogels possess good compliance, where the compression modulus is comparable to that of the silicone breast prosthesis (~23 kPa). Meanwhile, the hydrogels have an excellent self-recovery ability and fatigue resistance: the dissipative energy and elastic modulus recover almost completely after waiting for 2 min under cyclic compression, and the maximum strength remains essentially unchanged after 1000 cyclic compressions. More importantly, in vitro cellular experiments and in vivo animal experiments demonstrate that the hydrogels have good biocompatibility and stability. The biocompatible hydrogels with breast tissue-like mechanical properties hold great potential as an alternative implant material for reconstructing breasts.

## 1. Introduction

The breast is an important sign of female secondary sexual characteristics and physical beauty. However, due to female breast dysplasia, breast atrophy after breastfeeding, or breast defect after breast tumor resection, women lose physical beauty, leading to a series of psychological and social reactions [1,2,3]. To reconstruct the breast, many methods have been proposed, including prosthesis implantation, autologous tissue transplantation, and autologous tissue combined with prosthesis transplantation [4,5,6]. Although autologous tissue transplantation has outstanding effects such as a natural appearance and realistic feeling, this approach is at the expense of healthy tissue, which leads to many complications and additional damage [7,8]. Prosthesis implantation for breast reconstruction can avoid this problem. Meanwhile, the method has the characteristics of strong adaptability, a wide range of materials, high safety, high desirability, good compatibility, etc. Therefore, prosthesis implantation has been an important method for breast reconstruction.

The existing prosthesis mainly includes saline prosthesis and silicone prosthesis [9,10]. Compared to silicone prostheses, saline prostheses are prone to leakage and contamination, leading to the invalidation of breast reconstruction [11]. Therefore, silicone prostheses with breast tissue-like properties are extensively used in the clinical field. Although the silicone prosthesis is less likely to leak and contaminate, silicone can lead to regional lymphadenopathy (lymphadenitis is a nonspecific lymphadenitis caused by pathogenic bacteria invading lymphatic vessels from damaged and ruptured skin or mucosa through the lymphatic space of tissues and subsequently involving lymph nodes) and extramammary organ involvement if it leaks [12]. Therefore, it is necessary to develop a biocompatible prosthesis material that does not cause adverse reactions [13,14,15,16,17]. Hydrogels have a similar water content, porous structure, and good biocompatibility to biological tissue [18,19,20,21,22]. Thus, a prosthetic implant has a very promising application prospect. However, in the current study, the hydrogel materials cannot simultaneously meet the characteristics of a low modulus, high toughness, self-recovery property, and fatigue resistance for fat repair and regeneration. Therefore, it is urgent to propose a new design principle to prepare a breast tissue-like hydrogel for breast reconstruction.

Herein, we propose a strategy of hydrogen bond-reinforcing interactions and phase separation suppression to prepare a series of breast tissue-like PHEMA-based hydrogels. The hydrogels are fabricated by copolymerizing hydroxyethyl methacrylate (HEMA) and maleic acid (MA). MA units interact with HEMA units to not only form strong hydrogen bonds but also suppress the phase separation of PHEMA chains in the water-rich matrix. The unique network endows the hydrogels with good compliance, strength, and toughness. The hydrogels possess excellent mechanical properties under stretching deformation. The tensile strength, fracture strain, tensile modulus, and toughness are up to 420 kPa, 293.4%, 770 kPa, and 0.86 MJ/m^3^, respectively. Moreover, the hydrogels are quite compliant with a compression modulus in the range of 40–80 kPa, which displays the same order of magnitude of the compression modulus as the silicone breast prosthesis (SBP). In addition, after waiting for 2 min, the dissipated energy and elastic modulus of the hydrogels are almost completely recovered; the maximum strength of the hydrogels remains almost unchanged after 1000 cycles of compression, indicating their excellent self-recovery ability and fatigue resistance. More importantly, cell experiments in vitro and animal experiments in vivo proved that the hydrogels have good biocompatibility and stability. The biocompatible hydrogels with breast tissue-like mechanical properties hold great potential as an alternative implant material for breast reconstruction.

## 2. Results and Discussion 

### 2.1. Synthesis and Characterization of PHMx Hydrogels

**Figure 1:**
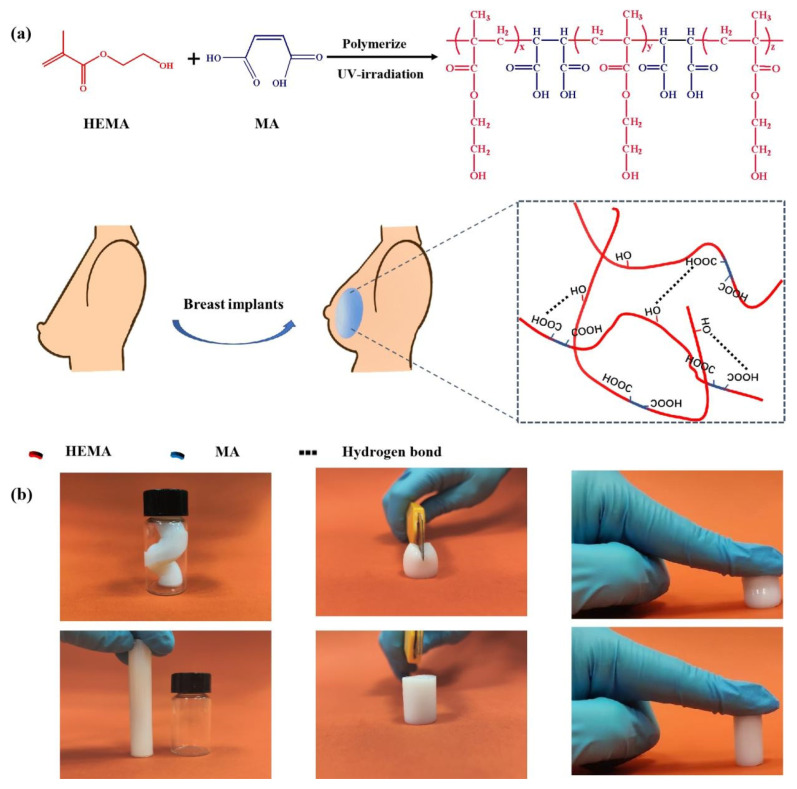
(**a**) Schematic diagram of the fabrication procedure of PHMx hydrogels. (**b**) The hydrogels can withstand a high degree of various mechanical deformations such as bending, cutting, and compressing.

In this study, we utilize MA units so as to have them interact with HEMA units by copolymerization in order to form hydrogen bonds (Figure 1a), resulting in an interaction reinforcement hydrogel network. At the same time, the hydrogen bonds between MA and HEMA units are stronger than those between HEMA and HEMA units, inhibiting the phase separation of PHEMA chains in the water-rich matrix. Moreover, due to the strong polarity and cis-substituted structure of MA, it cannot be homopolymerized with itself but copolymerized with the HEMA monomer [23]. Therefore, the MA unit dependently and homogeneously distributes in the molecular chains, providing uniform hydrogen bonding sites. As a result, a hydrogel network with robust hydrogen bonds is successfully constructed. As shown in Figure 1b, the hydrogel can maintain its shape after being highly bent, cut, and compressed, indicating its excellent mechanical properties. 

**Figure 2:**
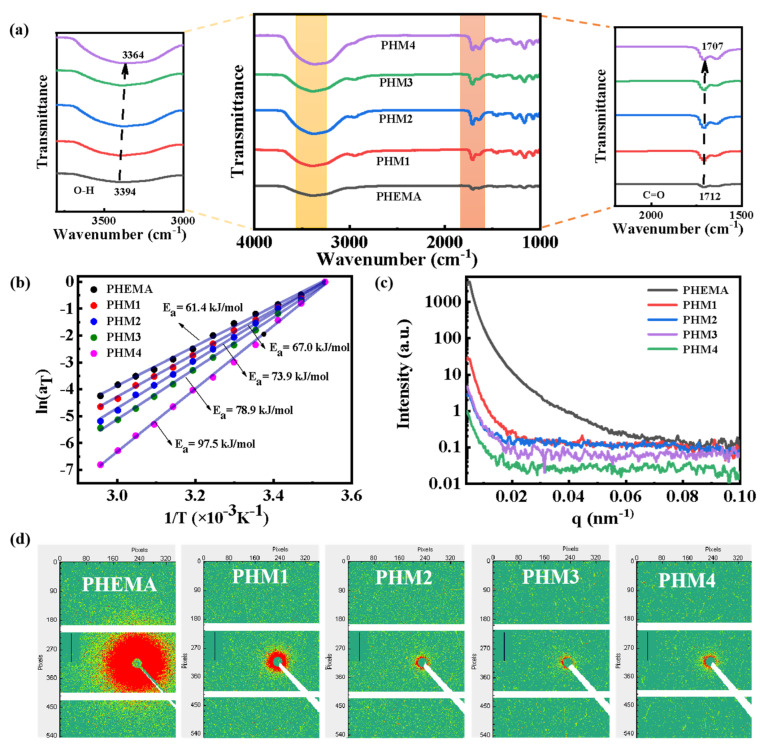
Structural characterization of the hydrogels. (**a**) FT−IR spectra of the PHEMA and PHMx hydrogels. (**b**) Arrhenius plot of horizontal displacement factor a_T_, and the apparent activation energy E_a_ determined by the slope of the diagram of a_T_. (**c**) 1D intensity distribution diagrams and (**d**) 2D SAXS diagrams of the PHEMA and PHMx hydrogels.

To analyze the structural characteristics of the hydrogels, FT-IR, rheological, and SAXS tests are carried out. As shown in Figure 2a, there are two peaks centered at 3394 and 1712 cm^−1^ in the PHEMA hydrogel, belonging to the O-H and C=O groups [24,25], respectively. As the MA content increases from 0 to 1.40 M, the characteristic peaks of O-H and C=O shift from 3394 and 1712 cm^−1^ to 3364 and 1707 cm^−^^1^, respectively. This result proves that hydrogen bonding interactions in the PHMx hydrogel become stronger as the MA content increases. Furthermore, the hydrogen bonding interactions are characterized by rheological tests through frequency scanning from 1–100 rad/s at temperatures ranging from 10 °C to 65 °C. Taking 10 °C as the reference temperature, the storage modulus G’, loss modulus G”, and loss factor tanδ curves at different temperatures are translated and superposed to obtain the main curves based on the time-temperature superposition principle (Figures S1–S5). The relationships between the horizontal displacement factor a_T_ and the temperature of the hydrogels are shown in Figure 2b. The apparent activation energy E_a_ of the PHMx hydrogel is much higher than that of the PHEMA hydrogel (61.4 kJ/mol). With the MA content increasing from 0.35 to 1.40 M, the apparent activation energy E_a_ increases from 67.0 to 97.5 kJ/mol, respectively. The above results confirm the formation of robust hydrogen bonds in the PHMx hydrogel, which is consistent with the results of FT-IR. In addition, after introducing MA units, the scattered intensity and signal decrease remarkably (Figure 2c,d). This shows that the aggregation of the PHEMA chains is significantly decreased by hydrogen-bonding interactions between the MA units and HEMA units [26,27], resulting in a homogeneous structure. With the MA content increasing, the scattering intensity and signal of the PHMx hydrogels decrease gradually, indicating that the phase separation is gradually inhibited and the network uniformity of the PHMx hydrogels is gradually improved. The above results show that a homogeneous hydrogel network with robust hydrogen bonds is successfully formed through the hydrogen bond-reinforcing interactions and phase separation inhibition. 

### 2.2. Mechanical Properties of the PHMx Hydrogels

**Figure 3:**
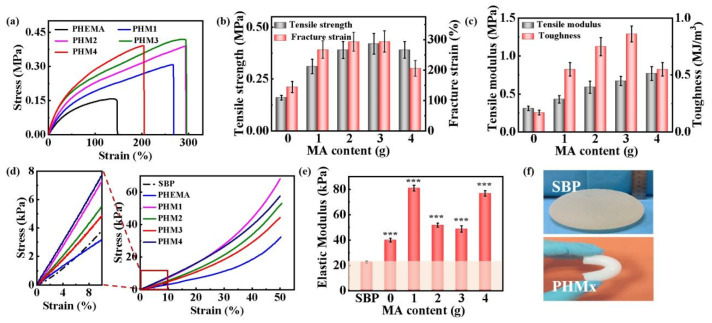
Mechanical properties of the hydrogels. (**a**) Tensile stress-strain curves of the PHMx hydrogels with different MA contents, and corresponding (**b**) tensile strength and fracture strain and (**c**) tensile modulus and toughness. (**d**) Compressive stress-strain curves of SBP and the PHMx hydrogels and (**e**) corresponding elastic modulus (NS represents no significant difference, * *p* < 0.05, ** *p* < 0.01, and *** *p* < 0.001). (**f**) Physical images of SBP and the PHMx hydrogels.

The unique network imparts the PHMx hydrogels with excellent mechanical properties. Compared to the tensile strength, fracture strain, tensile modulus, and fracture toughness of the PHEMA hydrogel, those of the PHMx hydrogels are significantly improved (Figure 3a–c). With the increase of the MA content, the tensile strength, fracture strain, and fracture toughness first increase and then decrease, reaching a maximum of 420 kPa, 293.45%, and 0.86 MJ /m^3^ with the addition of 1.05 M MA, respectively. The results demonstrate that the introduction of strong hydrogen bonds strengthens and toughens the PHMx hydrogels [28,29]. In addition to their excellent tensile properties, the hydrogels have good compression properties. The PHMx hydrogels possess a compression modulus of 40–80 kPa, displaying the same order of magnitude for the compression modulus as SBP (~23 kPa) (Figure 3d,e). The result shows that the hydrogels have a similar compliance to SBP under compression deformation. Overall, the PHMx hydrogels exhibit SBP-like mechanical properties (Figure 3f). Among the PHMx hydrogels, the PHM3 hydrogel shows the best comprehensive performance. Therefore, the PHM3 hydrogel is selected to study subsequent related properties. The reaction yield of PHM3 is 83.33%. 

### 2.3. Energy Dissipating Capability, Self-Recovery Property, and Fatigue Resistance of the PHMx Hydrogels

**Figure 4:**
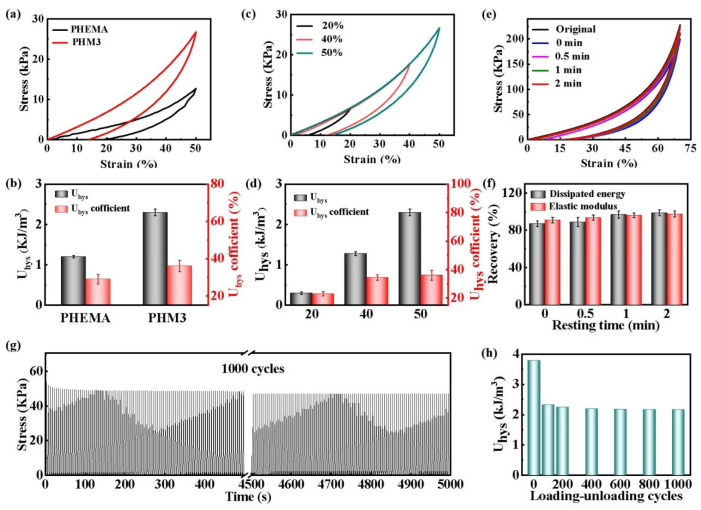
Energy dissipating capability, self-recovery property, and fatigue resistance of the PHMx hydrogels. (**a**) Compressive loading-unloading curves and (**b**) the dissipation energy U_hys_ and coefficient of U_hys_ of the PHEMA and PHM3 hydrogels at a maximum strain of 50%. (**c**) Compressive loading and unloading curves and (**d**) the dissipation energy U_hys_ and coefficient of U_hys_ of the PHM3 hydrogels at different maximum strains. (**e**) Cyclic tensile loading-unloading curves and (**f**) the recovery rate of the dissipation energy and elastic modulus for PHM3 hydrogels at different waiting times. (**g**) The variation of the maximum stress corresponds to 1000 times the continuous compression at the maximum strain of 50% and (h) the corresponding dissipation energy U_hys_.

The dynamical feature of the hydrogen bonds endows the PHMx hydrogels with an energy dissipating capability, self-recovery property, and fatigue resistance, which are essential for their long-term use as a breast prosthesis material. Loading-unloading cyclic compressive tests are performed on the PHEMA and PHM3 hydrogels. With the introduction of MA, the dissipation energy U_hys_ and the coefficient of U_hys_ of the PHM3 hydrogel increase from 1.2 kJ/m^3^ and 29% to 2.3 kJ /m^3^ and 63%, respectively (Figure 4a,b). Moreover, under different maximum strains, the dissipation energy U_hys_ and the coefficient of U_hys_ of the PHM3 hydrogel gradually increase from 0.3 kJ/m^3^ and 23% to 2.3 kJ/m^3^ and 36%, respectively (Figure 4c,d). The results indicate a good energy dissipating capability of the PHM3 hydrogel due to the strong hydrogen bonds.

Meanwhile, the hydrogels possess good self-recovery properties as well. The PHM3 hydrogel is subjected to continuous loading-unloading compression tests at a maximum strain of 70% with different waiting times. In the absence of a time interval, the dissipative energy and elastic modulus are significantly reduced (Figure 4e). As the waiting time increases, the broken hydrogen bonds are progressively re-formed, which makes the mechanical properties of the PHM3 hydrogel gradually recover. After waiting for 2 min, the elastic modulus and dissipated energy of the PHM3 hydrogel almost completely recover to the original state (Figure 4f). In addition, the PHM3 hydrogel maintains almost the same maximum stress, even after a periodic 50% strain at a high speed of 150 mm/min over 1000 cycles. The maximum stress slightly decreases from 53.8 kPa to 49.94 kPa after 1000 successive compressions (Figure 4g). The dissipation energy U_hys_ of the 1000th cycle is roughly the same as that of the 100th cycle (Figure 4h). The results indicate that the PHM3 hydrogel possesses excellent fatigue resistance and mechanical stability. The excellent fatigue resistance properties are attributable to a homogeneous structure with robust hydrogen bonding interactions. Based on these properties, the PHMx hydrogels are expected to be used as an alternative material for breast reconstruction.

### 2.4. Biocompatibility of the PHMx Hydrogels

**Figure 5:**
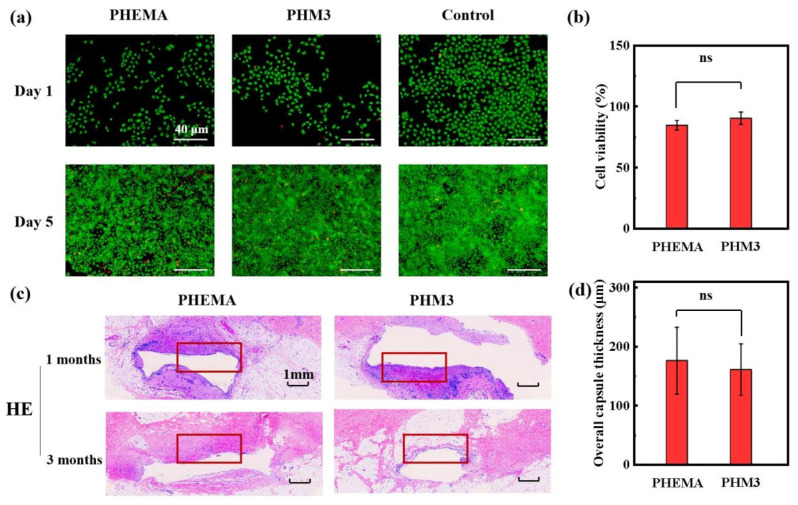
Biocompatibility of the hydrogels. (**a**) Representative fluorescence images of the PHEMA and PHM3 hydrogels after 1 and 5 days of cell culture. (**b**) Cell viability determined by the CCK method after 24 h. (**c**) Representative images of histological sections of explants implanted subcutaneously in swine for 1 and 3 months with H&E staining (the red rectangle indicates the capsule). (**d**) Overall capsule thickness of the PHEMA and PHM3 hydrogel samples implanted for 3 months (NS represents no significant difference).

A superior biocompatibility is necessary for breast prosthesis replacement materials. To evaluate the potential of the PHMx hydrogels as a breast prosthesis, a cell viability assay and cell live/dead assay are conducted to examine the cytocompatibility of the hydrogels. Taking the PHM3 hydrogel as an example, the live cell density of the PHEMA and PHM3 hydrogel groups is similar after culturing for 1 and 5 days (Figure 5a). The cell viability of the PHEMA and PHM3 hydrogel groups is 84.5% and 90.2%, respectively (Figure 5b). The cell viability of the PHM3 hydrogel is similar to that of the PHEMA hydrogel. The above results indicate that the good cytocompatibility of the PHEMA hydrogel is well maintained after the introduction of the MA units. Furthermore, the biocompatibility of the PHMx hydrogels is verified in in vivo experiments. The hydrogel samples are implanted subcutaneously in the male Bama minipig for 3 months. As shown in the H&E staining images, tissue bleeding and inflammatory cells near the samples decrease with the increase of the embedding time (Figure 5c). Meanwhile, the capsule thickness of the PHM3 hydrogel sample (160 μm) is even smaller than that of the PHEMA hydrogel sample (175 μm) (Figure 5d). The above results indicate that the PHMx hydrogels have an excellent histocompatibility. Therefore, the biocompatible hydrogels with breast tissue-like mechanical properties hold great potential as an alternative implant material for breast reconstruction.

## 3. Conclusions

In this work, a class of breast tissue-like PHEMA-based hydrogels is prepared by hydrogen bond-reinforcing interactions and phase separation suppression. The unique network fabricated by copolymerizing hydroxyethyl methacrylate (HEMA) and maleic acid (MA) endows the hydrogels with excellent compliance, strength, and toughness. The tensile strength, fracture strain, tensile modulus, and toughness are up to 420 kPa, 293.4%, 770 kPa, and 0.86 MJ/m^3^, respectively. Meanwhile, the hydrogels show the same order of magnitude for the compression modulus as SBP (~23 kPa), indicating their compliance. Moreover, under cyclic compression, the dissipated energy and elastic modulus of the hydrogels are almost entirely recovered after waiting for 2 min; after 1000 cyclic compression tests, the maximum strength of the hydrogel remains almost unchanged, which lays the foundation for the replacement of the PHMx hydrogel in the breast. In addition, the hydrogels also possess a good biocompatibility and stability, as demonstrated by in vitro cell experiments and in vivo animal experiments. Therefore, the PHMx hydrogels with combinational advantages are expected to become a breast prosthesis material.

## 4. Materials and Methods

### 4.1. Materials

Hydroxyethyl methacrylate (HEMA), maleic acid (MA), and deuterium oxide (D_2_O) were purchased from the Adamas-beta (Shanghai, China). 2-Hydroxy-2-methylpropiophenone (1173) was purchased from TCI (Shanghai, China). All reagents used in this study were used as received. Deionized water was used throughout this work.

### 4.2. Preparation of Poly(HEMA-co-MAx) (PHMx) Hydrogels

Firstly, a certain amount of monomer HEMA, MA, and photoinitiator 1173 were dissolved in deionized water, and then the mixed solution was deoxidized and irradiated for 2 h under 250 W UV to obtain pristine hydrogels. Then, the pristine hydrogels were immersed in deionized water for 24 h to remove unreacted reagents, obtaining equilibrium poly(HEMA-co-MAx) hydrogels, named PHMx hydrogels, where x represents the MA content. For comparison, the same amount of HEMA was polymerized under 250 W UV for 2 h and then immersed in deionized water for 24 h to prepare pure PHEMA hydrogel. The specific naming and formulation of the hydrogels are shown in Table 1.

### 4.3. Structural Characterization

Fourier transform infrared (FT-IR) measurements: The chemical structures of the PHM3 and PHEMA hydrogels were characterized by a VERTEX70 FT-IR spectrometer (Kono, America). The related samples and background were scanned 32 times with a scanning range of 4000~500 cm^−1^ and a resolution of 4 cm^−1^.

Gel permeation chromatography (GPC) measurements: The molecular weight of PHM3 and PHEMA was tested by a Tosoh HIC-8320 GPC (Japan). Tetrahydrofuran (THF) was used as the eluent. The number-average molecular weight (M_n_) and polydispersity of the PHEMA hydrogels were 94,434 and 1.03, respectively, and the number-average molecular weight (M_n_) and polydispersity of the PHM3 hydrogels were 91,401 and 1.05, respectively. 

Small-angle X-ray scattering (SAXS) measurements: SAXS tests were carried out by an MP-Xeuss 2.0 X-ray scatterometer, (Xenocs, Grenoble, France).

### 4.4. Rheological Measurements

Rheological tests were conducted on a TA AR2000ex rheometer (TA instrument Ltd., New Castle, DE, USA). The PHEMA and PHMx hydrogels were prepared in the form of disks (diameter: 40 mm, thickness: 2 mm). Frequency scanning tests were performed on the hydrogels at different temperatures ranging from 10 °C to 65 °C, frequencies ranging from 1–100 rad/s, and a strain fixed at 0.05%. Based on the time-temperature equivalence principle, the storage modulus G’, loss modulus G”, and loss factor tanδ curves were shifted at different temperatures to obtain the main curves of the rheological wide frequency range with a reference temperature of 10 °C. The horizontal displacement factor a_T_ of the time-temperature superposition conforms to the Arrhenius equation [27,30]:

(1) $$a_{T}=e^{-E_{a}/RT}$$

After taking the logarithm of both sides, the equation is transformed into:

(2) $$\ln a_{T}=-E_{a}/RT$$

where *E_a_*, *R*, and *T* represent the apparent activation energy, gas constant, and test temperature, respectively.

### 4.5. Mechanical Properties Measurements

The mechanical properties of the hydrogels were systematically tested by an Instron 3367 universal testing machine (Instron Corporation, Norwood, MA, USA). In tensile tests, dumbbell-shaped specimens (gauge length: 20 mm, width: 4 mm, thickness: 2 mm) are drawn at a rate of 100 mm/min until fracture, and the toughness is determined by the integral area under the stress-strain curve. In the compression tests, the cylindrical specimens (diameter: 12 mm, height: 18 mm) were compressed at a rate of 5 mm/min to a 50% strain. To evaluate the energy dissipation and self-recovery ability of the hydrogels, loading-unloading compression tests were carried out at a speed of 100 mm/min. The dissipated energy (U_hys_) is calculated from the area of the hysteresis loop. The coefficient of U_hys_ is determined by the ratio of the area of the lag circle to the area under the load curve. To evaluate the fatigue resistance of the hydrogels, 1000th cycles of loading-unloading compression tests were carried out with a strain of 50% and a rate of 150 mm/min.

### 4.6. In Vitro Cell Experiments

Cell viability assay: Human umbilical vein endothelial cells (HUVECs) purchased from the Cell bank of the Chinese Academy of Sciences (Shanghai, China) were seeded on 96-well plates with a density of 5000 cells per well. HUVECs were plated in DMEM-low glucose supplemented with FBS 10% and 1% PS, and incubated at 37 °C in 5% CO_2_. After 24 h of cultivation, the cells were co-cultured with the sterilized hydrogel sample for 24 h. Then, the hydrogel samples were removed and the culture media were sucked away. The CCK reagent was mixed with the culture medium in a volume ratio of 1:9. Add 10 µL of the mixture to each well, and incubate at 37 °C for 2 h. The absorbance at 450 nm was measured with a SynergyMx microplate reader (USA) to evaluate the cell activity. The value is expressed as the percentage of cells in the experimental group to the control group.

Cell live/dead assay: HUVECs were seeded on 24-well plates with a density of 2.4×10^5^ cells per well. After 24 h of culture, it was co-cultured with the sterile hydrogel for 1 and 3 days. Then, the hydrogel samples were removed and the cells were stained with Acridine Orange/Ethidium Bromide (AO/EB), in which living cells and dead cells were marked as green and red, respectively. Fluorescence images were acquired by a D1/AX10 cam HRC inverted fluorescence microscope (Zeiss, Dresden, Germany).

### 4.7. In Vivo Animal Experiments

To verify the biocompatibility of the material in vivo, in vivo experiments were carried out. Male Bama minipigs (5 months old, weighing 18 kg) were used in the experiments. The degree of inflammation was determined by staining the embedding site and observing its pericardial thickness. The pigs were anesthetized with Tiletamine-Zolazepam (5 mg/kg, ZoletilTM50, VIRBAC), and the PHEMA and PHMx hydrogel samples were implanted subcutaneously. After surgery, pigs were kept in cages alone for 1 and 3 months and given penicillin to reduce the risk of infection. Throughout the treatment period, the pigs did not experience any adverse reactions. The pigs were sacrificed 1 and 3 months after the operation, and the specimens were collected. The collected samples were fixed in 10% formalin buffer and then embedded with paraffin wax. Hematoxylin and Eosin staining (H&E) were used for the histological evaluation. Three fields were randomly selected for each section.

Statistical analysis: All data were obtained from at least three replicate trials and expressed as the mean ± standard deviation (SD). A one-way ANOVA after Tukey’s test was statistically analyzed by GraphPad Prism software. NS indicates a non-significant difference.

## Figures and Tables

**Table 1:** The composition of the hydrogels with different MA contents.

Samples	HEMA (M)	MA (M)	1173 (M)
PHEMA	2.50	0	0.01
PHM1	2.50	0.35	0.01
PHM2	2.50	0.70	0.01
PHM3	2.50	1.05	0.01
PHM4	2.50	1.40	0.01

## Data Availability

Not applicable.

## Supplementary Materials

The following supporting information can be downloaded at: https://www.mdpi.com/article/10.3390/gels8090532/s1, Figure S1: The main curves obtained by shifting the frequency sweep curves of the PHEMA hydrogel at different temperatures, where the reference temperature is 10 °C. Figure S2: The main curves obtained by shifting the frequency sweep curves of the PHM1 hydrogel at different temperatures, where the reference temperature is 10 °C. Figure S3. The main curves obtained by shifting the frequency sweep curves of the PHM2 hydrogel at different temperatures, where the reference temperature is 10 °C. Figure S4. The main curves obtained by shifting the frequency sweep curves of the PHM3 hydrogel at different temperatures, where the reference temperature is 10 °C. Figure S5: The main curves obtained by shifting the frequency sweep curves of the PHM4 hydrogel at different temperatures, where the reference temperature is 10 °C.
