# Supplementary material for: Compliant, Tough, Anti-Fatigue, Self-Recovery, and Biocompatible PHEMA-Based Hydrogels for Breast Tissue Replacement Enabled by Hydrogen Bonding Enhancement and Suppressed Phase Separation
Source: Gels. 2022 Aug 25;8(9):532. doi: 10.3390/gels8090532 (PMC9498755; doi:10.3390/gels8090532)
Supplement: Supplementary file 1 [file gels-08-00532-s001.zip › gels-1848483-supplementary.pdf]

# Compliant, tough, anti-fatigue, self-recovery, and biocompatible PHEMA-based hydrogels for breast tissue replacement enabled by hydrogen bonding enhancement and suppressed phase separation

**Hongyan Ouyang‡[1], Xiangyan Xie‡[1], Yuanjie Xie[1], Di Wu[1], Xingqi Luo[1], Jinrong Wu[2], Yi Wang[1]* and Lijuan Zhao[1]***

1 College of Chemistry and Materials Science, Sichuan Normal University, Chengdu 610068, China
2 State Key Laboratory of Polymer Materials Engineering, College of Polymer Science and Engineering, Sichuan University, Chengdu 610065, China
‡ These authors contributed equally to this paper.
* Correspondence: Dr. Yi Wang; College of Chemistry and Materials Science, Sichuan Normal University, China; Email: wangyi2020@sicnu.edu.cn; Dr. Lijuan Zhao; College of Chemistry and Materials Science, Sichuan Normal University, China; Email: lijuan_zhao@sicnu.edu.cn

**Contents**

**1. The main curves obtained by shifting the frequency sweep curves of the PHEMA hydrogel at different temperatures, where the reference temperature is 10 °C.**
**Figure S1 Page 2**
**2. The main curves obtained by shifting the frequency sweep curves of the PHM1 hydrogel at different temperatures, where the reference temperature is 10 °C.**
**Figure S1 Page 2**
**3. The main curves obtained by shifting the frequency sweep curves of the PHM2 hydrogel at different temperatures, where the reference temperature is 10 °C.**
**Figure S1 Page 3**
**4. The main curves obtained by shifting the frequency sweep curves of the PHM3 hydrogel at different temperatures, where the reference temperature is 10 °C.**
**Figure S1 Page 3**
**5. The main curves obtained by shifting the frequency sweep curves of the PHM4 hydrogel at different temperatures, where the reference temperature is 10 °C.**
**Figure S1 Page 4**

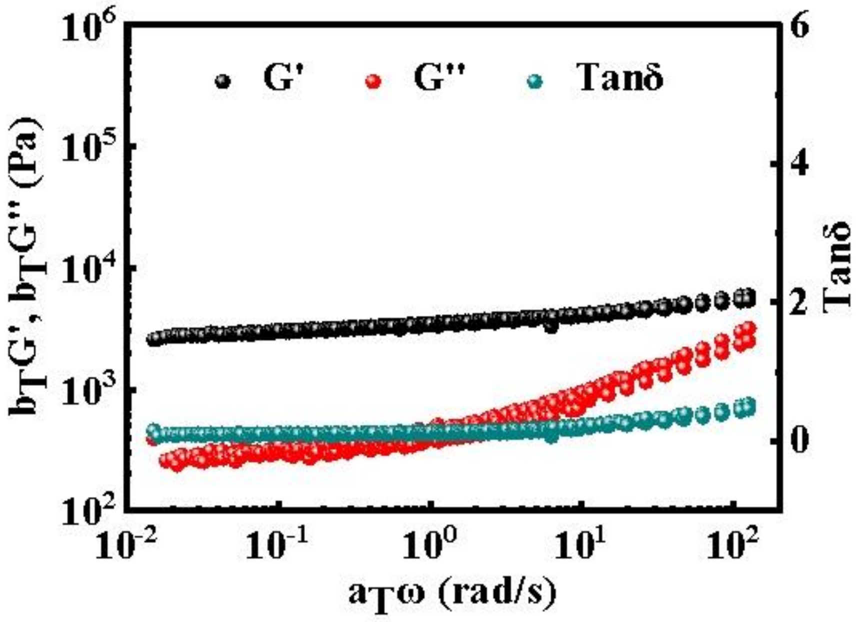


**Figure S1. The main curves obtained by shifting the frequency sweep curves of the PHEMA hydrogel at different temperatures, where the reference temperature is 10 °C.**

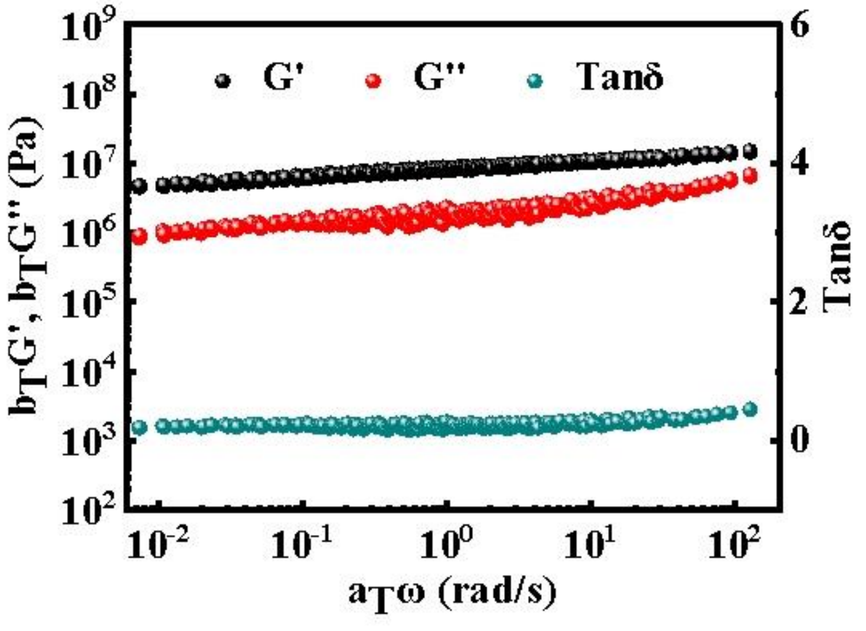


**Figure S2. The main curves obtained by shifting the frequency sweep curves of the PHM1 hydrogel at different temperatures, where the reference temperature is 10 °C.**

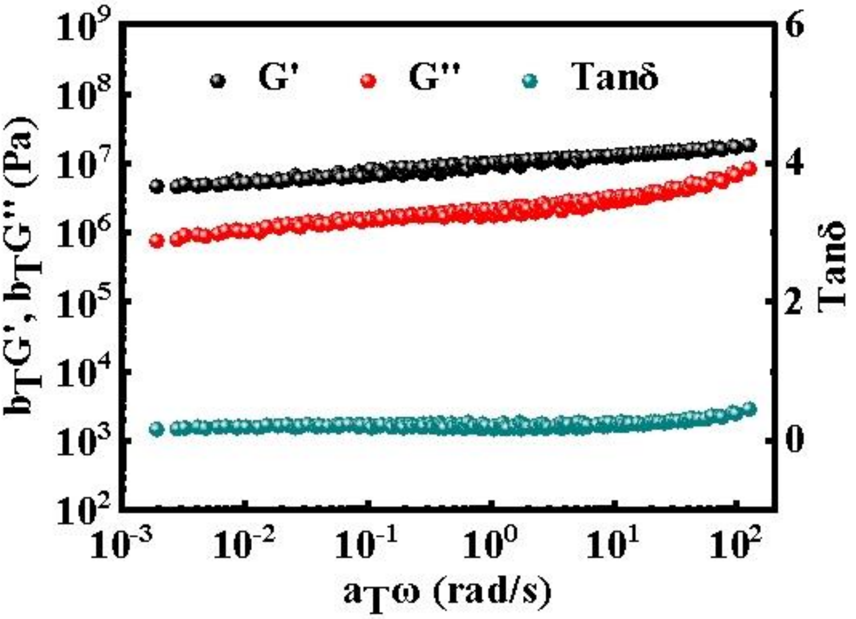


**Figure S3. The main curves obtained by shifting the frequency sweep curves of the PHM2 hydrogel at different temperatures, where the reference temperature is 10 °C.**

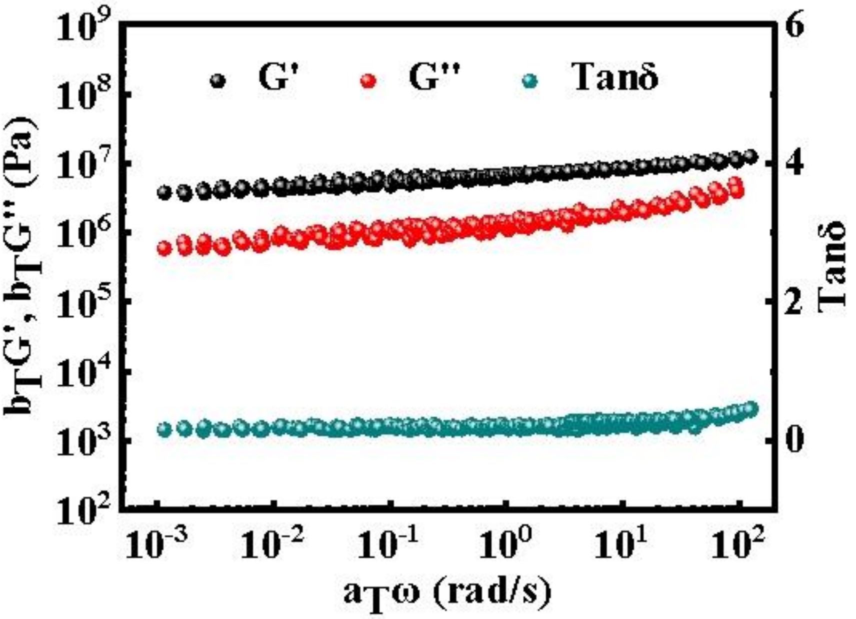


**Figure S4. The main curves obtained by shifting the frequency sweep curves of the PHM3 hydrogel at different temperatures, where the reference temperature is 10 °C.**

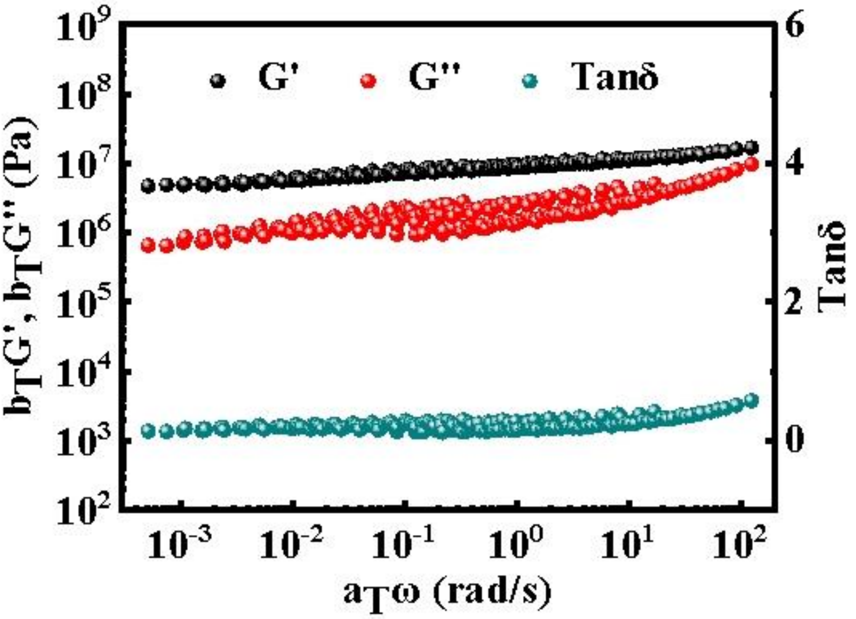


**Figure S5. The main curves obtained by shifting the frequency sweep curves of the PHM4 hydrogel at different temperatures, where the reference temperature is 10 °C.**
